# Relationship between physical activity and stiff or painful joints in mid-aged women and older women: a 3-year prospective study

**DOI:** 10.1186/ar2154

**Published:** 2007-03-29

**Authors:** Kristiann C Heesch, Yvette D Miller, Wendy J Brown

**Affiliations:** 1 School of Human Movement Studies, The University of Queensland, Blair Drive, Brisbane, Queensland 4072, Australia; 2 School of Psychology, The University of Queensland, Campbell Road, Brisbane, Queensland 4072, Australia

## Abstract

This prospective study examined the association between physical activity and the incidence of self-reported stiff or painful joints (SPJ) among mid-age women and older women over a 3-year period. Data were collected from cohorts of mid-age (48–55 years at Time 1; *n *= 4,780) and older women (72–79 years at Time 1; *n *= 3,970) who completed mailed surveys 3 years apart for the Australian Longitudinal Study on Women's Health. Physical activity was measured with the Active Australia questions and categorized based on metabolic equivalent value minutes per week: none (<40 MET.min/week); very low (40 to <300 MET.min/week); low (300 to <600 MET.min/week); moderate (600 to <1,200 MET.min/week); and high (1,200+ MET.min/week). Cohort-specific logistic regression models were used to examine the association between physical activity at Time 1 and SPJ 'sometimes or often' and separately 'often' at Time 2. Respondents reporting SPJ 'sometimes or often' at Time 1 were excluded from analysis. In univariate models, the odds of reporting SPJ 'sometimes or often' were lower for mid-age respondents reporting low (odds ratio (OR) = 0.77, 95% confidence interval (CI) = 0.63–0.94), moderate (OR = 0.82, 95% CI = 0.68–0.99), and high (OR = 0.75, 95% CI = 0.62–0.90) physical activity levels and for older respondents who were moderately (OR = 0.80, 95% CI = 0.65–0.98) or highly active (OR = 0.83, 95% CI = 0.69–0.99) than for those who were sedentary. After adjustment for confounders, these associations were no longer statistically significant. The odds of reporting SPJ 'often' were lower for mid-age respondents who were moderately active (OR = 0.71, 95% CI = 0.52–0.97) than for sedentary respondents in univariate but not adjusted models. Older women in the low (OR = 0.72, 95% CI = 0.55–0.96), moderate (OR = 0.54, 95% CI = 0.39–0.76), and high (OR = 0.61, 95% CI = 0.46–0.82) physical activity categories had lower odds of reporting SPJ 'often' at Time 2 than their sedentary counterparts, even after adjustment for confounders. These results are the first to show a dose–response relationship between physical activity and arthritis symptoms in older women. They suggest that advice for older women not currently experiencing SPJ should routinely include counseling on the importance of physical activity for preventing the onset of these symptoms.

## Introduction

Arthritis is a musculoskeletal condition of the joints. In Australia, it is a leading cause of pain and disability [1], affecting 3.4 million adults or 17% of the population [2]. Estimates are that by 2020 arthritis will affect 4.6 million Australians, or 20% of the adult population [2]. The current prevalence in Australia is slightly less than that in the United States, where 21% of the population has arthritis [3], making it the most prevalent chronic condition for mid-age and older people in the United States [4]. As in the United States, more Australian women than men have arthritis [2,4,5], and the incidence and prevalence of arthritis increase with age [4-6]. As the proportion of older people in both countries continues to rise, more individuals, particularly women, will be at risk of developing arthritis, and the burden of this disease will continue to increase. Identifying modifiable risk factors for the effects of arthritis is crucial to the prevention of its associated disability, especially in mid-age women and in older women.

Physical activity has been identified as a potentially modifiable risk factor in prospective population-based studies assessing risk factors for arthritis among women [5,7-9]. The results from these studies, however, are equivocal. One study [9] found walking to be protective against radiographic evidence of arthritis in women (defined as joint space narrowing), whereas others [5,7] found no association between leisure-time physical activity and risk of self-reported arthritis in women. In contrast, being in the highest quartile of total daily physical activity in the Framingham cohort study [8]* increased *the risk of incident radiographic arthritis in women in the short term (8 years), although not over a longer time period (20–40 years). Results of studies assessing risk factors for arthritis in male and female athletes indicate increased risk among competitive elite athletes in some sports, such as soccer, football, and rugby [10-13]. Together, the findings of these studies suggest that high levels of some competitive athletic sports increase the risk of arthritis but that moderate to vigorous leisure-time physical activities in nonathletes may have no association or reduce risk of the disease. Few studies have examined the association between physical activity and risk of arthritis in nonathletes, however, so this association is unclear.

The Australian Longitudinal Study on Women's Health (ALSWH) provides an opportunity to evaluate the prospective association between physical activity and increased risk of arthritis symptoms in two large cohorts of women. This prospective cohort study includes questions about walking and about moderate-intensity and vigorous-intensity physical activities. It also asks about physician diagnosis of arthritis and about women's experiences of a range of symptoms, including 'stiff or painful joints.' As there are more than 100 types of arthritis, all characterized by pain, stiffness, and disability [14], the self-report of these symptoms allows for the identification of women who have early and mild symptoms of arthritis, but have not yet been diagnosed with the disease. This is important because women with symptoms of arthritis do not always seek a professional diagnosis: estimates from the US National Health Interview Survey suggest that 16% of adults reporting arthritis have never seen a physician about this condition [15]. Indeed, many arthritis sufferers treat their symptoms with nonprescription medications or rely on alternative therapies [16-19]. There is also evidence to suggest that arthritis symptoms predict disability more strongly than radiological changes, which may not always be apparent in the early stages of the disease [20]. In exploring risk factors that contribute to the development of arthritis, the assessment of arthritis symptoms, therefore, may provide a more relevant and accurate indicator of the onset of the disease.

The aim of this study was to explore the association between physical activity and incidence of self-reported 'stiff or painful joints' in the mid-age and older cohorts of the ALSWH. Understanding the role of this potentially modifiable risk factor could be important in the development of strategies for the prevention of the disabling symptoms associated with arthritis in women.

## Materials and methods

### The ALSWH sample

The ALSWH is an ongoing study of the health and well-being of Australian women. As reported elsewhere [21], in 1996 random samples of women aged 18–23 years ('young'), 45–50 years ('mid-age'), and 70–75 years ('older') were drawn from the national Medicare health insurance database, which includes all Australian residents as well as immigrants and refugees. Women from rural and remote areas were intentionally over-represented. Data from the 2001 (Time 1 (T1)) and 2004 (Time 2 (T2)) surveys of the mid-age cohort and from the 1999 (T1) and 2002 (T2) surveys of the older cohort were used in the analyses reported here. The study was approved by the University of Newcastle Ethics Committee. Informed consent was received from all respondents. More details about the study can be found online [22].

### Assessment of stiff or painful joints

Respondents were asked whether they had experienced 'stiff or painful joints' in the past 12 months. Response options of 'never,' 'rarely,' 'sometimes,' or 'often' were dichotomized into 'sometimes or often,' or 'never or rarely' and also into 'often' or 'not often' (never, rarely, sometimes) to examine the sensitivity of the categorization chosen for determining the women at risk for incident joint pain. It was hypothesized that the women experiencing stiff or painful joints 'often' were those most likely to be suffering early symptoms of arthritis, and therefore physical activity would be more strongly associated with the onset of experiencing symptoms 'often' than 'sometimes or often.'

Because the validity of this item had not been examined, its predictive validity was assessed by exploring its ability to predict self-reported physician-diagnosed arthritis and physical functioning. Arthritis was assessed at T2 by asking 'In the last 3 years, have you been diagnosed with or treated for arthritis (including osteoarthritis, rheumatoid arthritis)?' [23]. Respondents who reported at T1 that they had been diagnosed with or treated for arthritis by a physician were excluded. In univariate logistic regression models, the odds of reporting arthritis at T2 were significantly increased among the mid-age women who reported stiff or painful joints 'sometimes or often' at T1 (odds ratio (OR) = 2.48, 95% confidence interval (CI) = 2.16–2.83, *P *< 0.001) and, similarly, among those who reported these symptoms 'often' (OR = 2.56, 95% CI = 2.13–3.09, *P *< 0.001). In the older women, reporting stiff or painful joints 'sometimes or often' also increased the odds of reporting arthritis (OR = 3.94, 95% CI = 3.38–4.58, *P *< 0.001), and reporting these symptoms 'often' increased the odds even more (OR = 5.28, 95% CI = 4.23–6.61, *P *< 0.001).

Physical function was measured with the Physical Function subscale of the Medical Outcomes Study Short Form [24]. A lower score on the subscale represents lower physical functioning. In univariate linear regression models, reporting stiff or painful joints 'sometimes or often' at T1 was associated with significantly lower physical function scores at T2 in both the mid-age women (*B *= -7.78, 95% CI = -8.58 to -6.99, *P *< 0.001) and older women (*B *= -14.15, 95% CI = -15.92 to -12.38, *P *< 0.001). Reporting the symptoms 'often' was associated with even lower physical function scores in the mid-age women (B = -14.37, 95% CI = -15.69 to -13.04, *P *< 0.001) and older women (*B *= -23.57, 95% CI = -26.42 to -20.73, *P *< 0.001).

### Assessment of physical activity

Survey items to assess physical activity were based on those developed for the Active Australia survey in 1997, a validated and reliable measure [25-27]. The frequency and time duration (in at least 10-min sessions) in the previous week spent walking briskly (for travel or leisure), in moderate-intensity leisure-time physical activities, and in vigorous leisure-time physical activities were reported. A physical activity score was calculated as the sum of the products of total time in each of the three categories of activity and the metabolic equivalent value (MET) assigned to each category [28,29]: (walking minutes × 3.0 METs) + (moderate physical activity minutes × 4.0 METs) + (vigorous physical activity minutes × 7.5 METs), in accordance with the Compendium of Physical Activities [30]. Physical activity was then categorized based on total MET minutes per week: none (<40 MET.min/week); very low (40 to <300 MET.min/week); low (300 to <600 MET.min/week); moderate (600 to <1,200 MET.min/week); and high (1,200+ MET.min/week).

### Assessment of potential confounding factors

A list of variables considered potential confounders in the relationship between physical activity and stiff or painful joints was derived from previous studies [31] (see Table 1). Area of residence categories were derived from postcodes. To measure the number of chronic diseases, respondents were asked whether they had been told by a doctor in the previous 3 years that they had any of the diseases listed. The list of diseases was adapted from the Australian 1989–1990 National Health Survey [23]. Diagnosis of depression was determined by a single item modified from the Australian 1989–1990 National Health Survey [23]: 'In the last 3 years, have you been told by a doctor that you have depression?' ('yes' or 'no').

**Table 1:** Characteristics of respondents who reported stiff or painful joints 'never' or 'rarely' at Time 1

	Mid-age women (*n *= 5,650)			Older women (*n *= 5,207)		
	
Variable	Respondents included (*n *= 4,780)	Respondents excluded^a ^(*n *= 870)	*P *value^b^	Respondents included (*n *= 3,970)	Respondents excluded^a ^(*n *= 1,237)	*P *value^b^
Age (years, mean ± standard deviation)	52.53 ± 1.49	52.57 ± 1.52	0.366	75.39 ± 1.51	75.60 ± 1.51	<0.001
Education (%)			<0.001			<0.001
Less than high school	13.5	18.7		26.8	34.7	
Some high school	47.8	50.9		52.7	47.7	
Completed high school	20.5	17.4		11.6	9.2	
Trade certificate/university degree	17.4	12.0		4.7	2.3	
Missing	0.9	1.0		4.3	6.1	
Area of residence (%)			<0.001			<0.001
Urban	38.1	43.3		40.2	39.8	
Large town	13.5	11.4		11.6	14.1	
Small town/remote area	47.1	42.4		46.6	42.6	
Missing	1.3	2.9		1.6	3.6	
Country of birth (%)			0.001			0.003
Australia	74.6	70.9		74.7	71.9	
Other English-speaking	14.0	12.9		12.4	11.2	
Non-English speaking	7.9	12.1		6.8	9.3	
Missing	3.5	4.1		6.0	7.7	
Depression (%)			0.023			<0.001
No	91.6	89.2		94.3	87.6	
Yes	8.4	10.8		3.4	7.6	
Number of chronic diseases (%)			0.037			<0.001
0	55.8	52.6		32.0	42.5	
1	31.0	30.5		37.0	20.3	
2	9.7	11.8		20.0	18.0	
3	2.7	3.7		7.6	10.5	
4 or more	0.8	1.4		3.3	8.7	
Smoking status (%)			<0.001			0.006
Never	55.4	54.8		61.0	58.5	
Former	32.2	26.1		27.6	26.8	
Current	12.2	18.3		4.9	7.4	
Missing	0.2	0.8		6.4	7.4	
Body mass index (%)			<0.001			<0.001
<20 kg/m^2^	5.1	5.9		3.4	4.4	
≥ 20 and <25 kg/m^2^	41.9	38.6		48.4	46.1	
≥ 25 and <30 kg/m^2^	28.0	26.3		26.5	23.8	
≥ 30 kg/m^2^	17.4	16.8		9.7	9.1	
Missing	7.5	12.4		12.0	16.6	
Physical activity (%)			<0.001			<0.001
None (<40 MET.min/week)	14.9	22.2		24.4	40.1	
Very low (40 to <300 MET.min/week)	18.4	19.5		14.0	14.2	
Low (300 to <600 MET.min/week)	18.0	15.6		22.7	14.0	
Moderate (600 to <1,200 MET.min/week)	22.5	19.6		15.8	12.2	
High (1,200+ MET.min/week)	26.2	23.1		23.1	19.6	

MET, metabolic equivalent value. ^a^Women were excluded if they did not provide data on physical activity at Time 1 or did not provide data on symptoms of stiff or painful joint at Time 2. The 243 mid-age women and 987 older women who were missing physical activity data are not included in the percentage of excluded respondents in each physical activity category. ^b^*P *value is for the difference between women included and those excluded from the analysis.

Height without shoes and weight without clothes or shoes were reported, and the body mass index (BMI) was calculated as weight divided by height squared. The BMI was then categorized as underweight (BMI <20 kg/m^2^), healthy weight (BMI ≥20 and <25 kg/m^2^), overweight (BMI ≥25 and <30 kg/m^2^), or obese (BMI ≥ 30 kg/m^2^) in accordance with the Australian National Health and Medical Research Council classification system [32]. The World Health Organization classification of a BMI less than 18.5 kg/m^2 ^as 'underweight' [33] was not used because few in the samples had a BMI meeting this criterion at the first ALSWH survey.

### Data analysis

The initial analysis samples were mid-age women and older women who did not report having stiff or painful joints 'sometimes' or 'often' at T1. From this group, respondents were excluded if they had missing physical activity data at T1 or had missing stiff or painful joint data at T2. Differences between women included in our analysis and those excluded were examined using Pearson's chi-square tests for categorical variables and an independent *t *test for the one continuous variable (age). Univariate associations between each potential confounding variable at T1 and the two outcomes (having stiff or painful joints 'sometimes or often;' having these symptoms 'often') at T2 were computed separately for each cohort. Variables having a statistically significant association with at least one outcome in at least one cohort (*P *< 0.05) were included in multivariable logistic regression models computed to evaluate the association between physical activity and stiff or painful joints in each cohort, after adjusting for the other factors. For each confounding variable for which some respondents' data were missing, a missing category was included in all analyses to maintain as large a sample as possible, and the missing category was compared with the reference category in the same way the other categories were compared with the reference category. Interactions between physical activity and each potential confounding variable were examined, but none were significant. No interaction terms were therefore included in the final models. Odds ratios and 95% confidence intervals were computed for all models.

## Results

### Samples

In total, 5,650 (52.2%) mid-age women and 5,207 (54.9%) older women reported having stiff or painful joints 'never' or 'rarely' at T1. Of these, 475 mid-age women and 843 older women were excluded because they did not participate in the T2 survey. Another 208 mid-age women and 199 older women were excluded because they had missing values for physical activity at T1. After the additional exclusion of women who did not report whether they had painful or stiff joints at T2 (187 mid-age women and 195 older women excluded), data from 4,780 mid-age women and 3,970 older women were included in these analyses.

Meaningful and statistically significant differences were seen between those who were included and those who were excluded from the analysis (see Table 1). In both cohorts, women who were excluded from the analysis were less physically active and had lower levels of education (*P *< 0.001). These women were also were more likely to live in a large town, to have been born in a non-English-speaking country, to have four or more chronic diseases, and to be smokers than women who were included (*P *< 0.05). Older women who were excluded were also more likely to have depression (*P *< 0.001).

### Descriptive characteristics of samples

The mid-age women were aged 48–55 years at T1. Most reported not completing 12 years of high school, reported living in a small rural town or remote area, reported being born in Australia, reported having one or no chronic diseases, reported not having a diagnosis of depression, and reported never having been a smoker. Almost one-half were overweight or obese (45.4%), and almost one-half (48.7%) met the national Australian physical activity guidelines by accruing 600 or more MET minutes of physical activity per week [34], which is equivalent to 150 minutes or more per week of moderate-intensity physical activity. Slightly more than one-third (36.4%) reported very low to low levels of physical activity (40–600 MET.min/week), which equates to 10–149 minutes per week of moderate-intensity physical activity. The remaining 14.9% were sedentary (<40 MET.min/week): they did not report even 10 minutes of moderate-intensity physical activity per week. At T2, 41.4% of the women reported 'never' having stiff or painful joints, 17.9% reported them 'rarely,' 30.8% reported them 'sometimes,' and 9.9% reported them 'often.'

The older women were aged 72–79 years at T1. As for the mid-age women, most reported not completing 12 years of high school, reported living in a small rural town or remote area, reported being born in Australia, reported not having a diagnosis of depression, reported having one or no chronic diseases, and reported never having been a smoker. Fewer older women (36.2%) than mid-age women were overweight or obese, and fewer were physically active. Less than one-half of the older women met the national physical activity guidelines (38.9%), and a similar percentage (38.7%) reported very low to low levels of physical activity. One-quarter (24.4%) of the older women were sedentary. At T2, 45.9% reported stiff or painful joints 'never', 12.2% reported them 'rarely,' 30.0% reported them 'sometimes,' and 11.8% reported them 'often.'

### Mid-age women

In univariate analysis, the odds of reporting stiff or painful joints 'sometimes or often' at T2 were significantly lower for mid-age women in the 'low' (*P *= 0.011), 'moderate' (*P *= 0.043), and 'high' (*P *= 0.003) physical activity categories at T1 than for those who were sedentary (see Table 2). The odds of reporting stiff or painful joints 'often' were significantly lower only for respondents in the 'moderate' physical activity category (*P *= 0.032). After adjusting for all variables that were significantly associated with stiff or painful joints in the univariate analyses, associations between physical activity and self-reported stiff or painful joints in the mid-age women were attenuated and no longer statistically significant (*P *> 0.05; see Table 2).

**Table 2:** Association between risk factors and having stiff or painful joints among mid-age women (*n *= 4,780)

	Stiff or painful joints 'sometimes or often'		Stiff or painful joints 'often'	
	
Variable at Time 1	Unadjusted odds ratio (95% confidence interval)	Adjusted^a ^odds ratio (95% confidence interval)	Unadjusted odds ratio (95% confidence interval)	Adjusted^a ^odds ratio (95% confidence interval)
Education				
Less than high school	1.00	1.00	1.00	1.00
Some high school	0.77 (0.65–0.92)	0.83 (0.69–0.99)	0.55 (0.43–0.71)	0.58 (0.45–0.75)
Completed high school	0.73 (0.60–0.90)	0.80 (0.65–0.99)	0.50 (0.37–0.68)	0.55 (0.40–0.76)
Trade certificate/university degree	0.64 (0.52–0.78)	0.70 (0.56–0.87)	0.49 (0.35–0.67)	0.55 (0.39–0.77)
Missing	0.97 (0.51–1.82)	0.92 (0.48–1.75)	1.51 (0.70–3.26)	1.30 (0.58–2.93)
Area of residence				
Urban	1.0	1.0	1.0	1.0
Large town	0.87 (0.73–1.05)	0.87 (0.72–1.05)	0.8 (0.58–1.11)	0.77 (0.55–1.07)
Small town/remote area	1.11 (0.98–1.26)	1.09 (0.96–1.24)	1.14 (0.93–1.39)	1.08 (0.88–1.34)
Missing	0.83 (0.49–1.40)	0.83 (0.49–1.42)	0.3 (0.75–1.28)	0.32 (0.76–1.33)
Country of birth				
Australia	1.00	1.00	1.00	1.00
Other English-speaking	1.07 (0.91–1.27)	1.12 (0.95–1.33)	0.70 (0.51–0.95)	0.70 (0.51–0.97)
Non-English speaking	0.97 (0.78–1.21)	1.02 (0.82–1.28)	0.96 (0.67–1.36)	0.99 (0.69–1.43)
Missing	1.35 (0.99–1.84)	1.36 (0.99–1.88)	1.64 (1.06–2.53)	1.61 (1.02–2.53)
Depression				
No	1.00	1.00	1.00	1.00
Yes	1.56 (1.29–1.94)	1.44 (1.17–1.78)	2.10 (1.60–2.77)	1.76 (1.32–2.35)
Number of chronic diseases				
0	1.00	1.00	1.00	1.00
1	1.41 (1.24–1.61)	1.35 (1.18–1.54)	1.78 (1.43–2.20)	1.62 (1.30–2.02)
2	1.54 (1.26–1.89)	1.37 (1.11–1.67)	2.67 (2.01–3.54)	2.17 (1.61–2.91)
3	1.93 (1.35–2.75)	1.67 (1.17–2.40)	2.53 (1.55–4.14)	1.96 (1.18–3.25)
4 or more	1.47 (0.77–2.82)	1.10 (0.56–2.14)	3.04 (1.32–7.01)	1.89 (0.79–4.49)
Smoking status				
Never	1.00	1.00	1.00	1.00
Former	1.00 (0.88–1.14)	0.99 (0.87–1.12)	1.23 (1.00–1.54)	1.21 (0.97–1.50)
Current	1.14 (0.95–1.36)	1.08 (0.90–1.30)	1.44 (1.09–1.91)	1.35 (1.01–1.81)
Missing	2.23 (0.63–7.91)	2.11 (0.59–7.60)	2.56 (0.54–12.10)	2.70 (0.55–13.2)
Body mass index				
<20 kg/m^2^	1.03 (0.79–1.36)	1.03 (0.78–1.36)	1.22 (0.76–1.95)	1.25 (0.78–2.01)
≥ 20 and <25 kg/m^2^	1.00	1.00	1.00	1.00
≥ 25 and <30 kg/m^2^	1.10 (0.96–1.27)	1.06 (0.92–1.23)	1.46 (1.15–1.86)	1.36 (1.06–1.74)
≥ 30 kg/m^2^	1.63 (1.38–1.92)	1.46 (1.23–1.73)	2.22 (1.73–2.86)	1.83 (1.41–2.38)
Missing	1.32 (1.05–1.66)	1.29 (1.02–1.62)	1.43 (0.98–2.08)	1.35 (0.92–2.00)
Physical activity				
None (<40 MET.min/week)	1.00	1.00	1.00	1.00
Very low (40 to <300 MET.min/week)	0.86 (0.71–1.05)	0.93 (0.76–1.14)	0.92 (0.67–1.26)	1.08 (0.78–1.49)
Low (300 to <600 MET.min/week)	0.77 (0.63–0.94)	0.88 (0.71–1.08)	0.87 (0.63–1.19)	1.15 (0.82–1.60)
Moderate (600 to <1,200 MET.min/week)	0.82 (0.68–0.99)	0.94 (0.77–1.14)	0.71 (0.52–0.97)	0.91 (0.66–1.27)
High (1,200+ MET.min/week)	0.75 (0.62–0.90)	0.88 (0.72–1.06)	0.78 (0.58–1.05)	1.06 (0.78–1.45)

^a^Adjusted for all other variables in the table.

### Older women

In univariate analysis, older women in the 'moderate' (*P *= 0.033) and 'high' (*P *= 0.040) physical activity categories at T1 had significantly lower odds of reporting stiff or painful joints 'sometimes or often' at T2 than those in the 'none' category. Significantly lower odds of reporting stiff or painful joints 'often' were found for those in the 'low' (*P *= 0.001), 'moderate' (*P *< 0.001) and 'high' (*P *< 0.001) physical activity categories (see Table 3).

**Table 3:** Association between risk factors and having stiff or painful joints among older women (*n *= 3,970)

	Stiff or painful joints 'sometimes or often' at Time 2		Stiff or painful joints 'often' at Time 2	
	
Variable at Time 1	Unadjusted odds ratio (95% confidence interval)	Adjusted^a ^odds ratio (95% confidence interval)	Unadjusted odds ratio (95% confidence interval)	Adjusted^a ^odds ratio (95% confidence interval)
Education				
Less than high school	1.00	1.00	1.00	1.00
Some high school	0.89 (0.76–1.04)	0.90 (0.76–1.05)	0.86 (0.68–1.09)	0.90 (0.71–1.16)
Completed high school	0.92 (0.74–1.13)	0.97 (0.78–1.20)	1.06 (0.77–1.44)	1.17 (0.85–1.62)
Trade certificate/university degree	1.01 (0.83–1.23)	1.06 (0.86–1.30)	0.80 (0.59–1.10)	0.93 (0.67–1.28)
Missing	0.89 (0.64–1.24)	0.91 (0.64–1.29)	1.25 (0.79–1.97)	1.37 (0.84–2.22)
Area of residence				
Urban	1.00	1.00	1.00	1.00
Large town	0.94 (0.76–1.16)	0.91 (0.73–1.13)	0.94 (0.67–1.31)	0.88 (0.62–1.24)
Small town/remote area	1.04 (0.91–1.19)	1.02 (0.89–1.18)	1.20 (0.98–1.48)	1.15 (0.93–1.42)
Missing	0.72 (0.42–1.22)	0.75 (0.43–1.29)	0.41 (0.13–1.32)	0.41 (0.12–1.33)
Country of birth				
Australia	1.00	1.00	1.00	1.00
Other English-speaking	0.95 (0.78–1.15)	0.93 (0.76–1.14)	0.87 (0.64–1.18)	0.90 (0.65–1.23)
Non-English speaking	1.00 (0.78–1.29)	0.92 (0.71–1.20)	1.02 (0.70–1.49)	0.90 (0.60–1.34)
Missing	0.94 (0.72–1.23)	0.94 (0.70–1.27)	1.02 (0.68–1.52)	0.91 (0.58–1.42)
Depression				
No	1.00	1.00	1.00	1.00
Yes	1.48 (1.04–2.09)	1.29 (0.90–1.84)	2.15 (1.41–3.29)	1.75 (1.13–2.72)
Number of chronic diseases				
0	1.00	1.00	1.00	1.00
1	1.26 (1.08–1.48)	1.23 (1.05–1.44)	1.42 (1.09–1.85)	1.37 (1.05–1.79)
2	1.90 (1.59–2.28)	1.83 (1.52–2.19)	2.09 (1.57–2.77)	1.93 (1.44–2.57)
3	2.43 (1.89–3.14)	2.33 (1.80–3.02)	2.83 (1.99–4.03)	2.53 (1.77–3.63)
4 or more	3.06 (2.12–4.43)	2.93 (2.02–4.26)	5.02 (3.28–7.69)	4.24 (2.74–6.57)
Smoking status				
Never	1.00	1.00	1.00	1.00
Former	1.07 (0.93–1.24)	1.08 (0.93–1.25)	1.22 (0.99–1.52)	1.27 (1.01–1.59)
Current	1.05 (0.78–1.40)	1.10 (0.81–1.49)	1.17 (0.76–1.82)	1.17 (0.75–1.84)
Missing	1.01 (0.77–1.31)	1.04 (0.78–1.37)	1.06 (0.71–1.59)	1.07 (0.70–1.64)
Body mass index				
<20 kg/m^2^	1.04 (0.72–1.48)	0.97 (0.67–1.39)	0.98 (0.54–1.77)	0.86 (0.47–1.58)
≥ 20 and <25 kg/m^2^	1.00	1.00	1.00	1.00
≥ 25 and <30 kg/m^2^	1.46 (1.26–1.70)	1.39 (1.19–1.63)	1.46 (1.15–1.84)	1.33 (1.04–1.68)
≥ 30 kg/m^2^	1.42 (1.14–1.77)	1.26 (1.00–1.58)	1.68 (1.23–2.31)	1.32 (0.95–1.84)
Missing	1.13 (0.92–1.39)	1.07 (0.87–1.32)	1.52 (1.13–2.05)	1.36 (1.00–1.85)
Physical activity				
None (<40 MET.min/week)	1.00	1.00	1.00	1.00
Very low (40 to <300 MET.min/week)	0.98 (0.80–1.22)	1.04 (0.84–1.29)	0.87 (0.65–1.17)	0.94 (0.70–1.27)
Low (300 to <600 MET.min/week)	1.00 (0.83–1.20)	1.11 (0.92–1.34)	0.63 (0.48–0.82)	0.72 (0.55–0.96)
Moderate (600 to <1,200 MET.min/week)	0.80 (0.65–0.98)	0.89 (0.72–1.10)	0.48 (0.34–0.67)	0.54 (0.39–0.76)
High (1,200+ MET.min/week)	0.83 (0.69–0.99)	0.94 (0.78–1.14)	0.51 (0.38–0.68)	0.61 (0.46–0.82)

^a^Adjusted for all other variables in the table.

As was the case for the mid-age women, the association between physical activity and self-reported stiff or painful joints 'sometimes or often' was no longer statistically significant (*P *= 0.252) in the multivariable analysis in the older cohort. The odds for reporting stiff or painful joints 'often,' however, remained significantly lower for older women in the 'low' (*P *= 0.024), 'moderate' (*P *< 0.001) and 'high' (*P *= 0.001) physical activity categories than for those in the 'none' category (see Table 3).

## Discussion

Our aim was to explore the association between physical activity and the incidence of stiff or painful joints in cohorts of mid-age women and older women. Our main findings were that physical activity did not increase or decrease the odds of self-reported stiff or painful joints 'often' among the mid-age women; however, 'low,' 'moderate,' and 'high' levels of physical activity among the older women were associated with decreased odds of developing stiff or painful joints 'often' over 3 years, even after adjusting for confounding variables. This last finding indicates that, among older women who do not have or rarely have stiff or painful joints, participation in at least 75 minutes per week of moderate-intensity physical activity may be protective against complaints of 'often' having arthritis symptoms within the next 3 years. The results also suggest that engaging in at least 150 minutes of moderate-intensity physical activity per week, in accordance with the recommendations of the American College of Sports Medicine and the US Centers for Disease Control and Prevention [35], may be even more protective. These findings consequently indicate that public health and clinical advice for older women not currently experiencing stiff or painful joints should routinely include counseling on ways to be physically active to reduce their risk of developing stiff or painful joints.

Different findings between the two ALSWH cohorts with respect to the relationship between physical activity and stiff or painful joints 'often' were unexpected. One explanation is that occupational physical activity was not included in our assessment of physical activity and that many women in the mid-age cohort of the ALSWH were in paid work [36], whereas the older women were not. Failure to account for occupational physical activity may have resulted in greater misclassification of physical activity levels among the mid-age women than among the older women, which might explain the difference in findings between the two cohorts. Researchers who have used a crude measure of work-related physical activity have not, however, found a prospective association between occupational physical activity and arthritis in women [9]. More precise measures of occupational physical activity are required to further explore these associations.

We did not observe a statistically significant association between physical activity and self-reported stiff or painful joints 'sometimes or often' in either cohort. This finding may reflect a wider variability in interpretation of the phrase 'sometimes' than 'often,' with some respondents exaggerating the frequency of their symptoms by selecting 'sometimes' when symptoms occurred 'rarely,' resulting in a weakened ability to detect an association.

The present study was the first to assess the prospective association between physical activity and symptoms of arthritis in two different age cohorts of women. Our observation of no statistically significant associations in three of the four multivariable analyses supports the results of prospective studies that have assessed the long-term associations between physical activity and arthritis in other large cohorts of women [5,7]. In a 25-year cohort study that included 4,073 women 20–87 years of age, Cooper Clinic (US) researchers [7] reported no statistically significant association between walking or jogging and self-reported physician-diagnosed hip and knee osteoarthritis for women after controlling for BMI, alcohol, smoking status, and caffeine consumption. In the 20-year Alameda County Cohort Study (US) [5], no statistically significant association between leisure-time physical activity and self-reported arthritis was seen among the 1,148 women who participated (mean age = 43 years for all participants) after controlling for age, race, BMI, and the presence of five or more depressive symptoms. Assessment of the risk factors for radiographic knee osteoarthritis among 715 mid-age women (aged 54 ± 6 years) in the Chingford Study Cohort (UK) [9] revealed that walking, occupational physical activity, and sport were not statistically significantly associated with incident osteophytes over 4 years after adjusting for age, social class, BMI, and smoking status among other factors – only walking was associated with decreased odds of joint space narrowing (OR = 0.38, 95% CI = 0.15–0.93) over that same time period after adjusting for the same variables.

Our finding that physical activity is protective against complaints of stiff or painful joints 'often' in older women does not support the results from these other studies [5,7-9]. Only the Framingham Study [8], however, focused specifically on older women. In that study, the researchers found an *increased *risk of radiographic knee osteoarthritis over 10 years (but not after 20 or 40 years) among the 69 older women (mean age = 71 ± 5 years for the sample of men and women) in the highest quartile of physical activity in a model adjusted for age, BMI, cigarette smoking, and other covariates (OR = 3.1, 95% CI = 1.1–8.6). In contrast, our results showed a clear dose–response relationship between physical activity and incident stiff or painful joint 'often' over 3 years in women aged 72–79 years at T1.

Interpretation of our results in the context of the findings from other studies should be made with caution because each study of the risk factors for arthritis has used a different measure of physical activity. In our study, a generic physical activity score reflected participation in walking as well as moderate-intensity and vigorous-intensity leisure-time activities during the past week, whereas other studies have used 24-hour recall [8], have focused on specific physical activities, such as walking [7,9], or have used their own physical activity index to evaluate habitual leisure-time physical activity [5]. Moreover, the outcomes of each study differed. While our study examined arthritis symptoms, other studies assessed self-reported arthritis [5], self-reported osteoarthritis [7], or radiographic osteoarthritis [8,9]. It should also be noted that different studies used follow-up periods ranging from 4 to 40 years [5,7-9]. Although our follow-up period of 3 years was short, it was appropriate for assessing the development of symptoms of arthritis rather than arthritis itself, which can take much longer to develop.

Our study does not provide insight into the mechanisms by which physical activity may impact development of arthritis symptoms in older women; however, the constellation of significant factors (physical activity, BMI, and smoking) supports the suggestion that there is a metabolic basis to the development of arthritis [9]. Alternatively, the links between physical activity and arthritis symptoms might be explained by exercise-related endorphin release, by protection against fibromyalgia, by increased resistance to musculoskeletal injury, by differences in pain threshold for people who exercise regularly, or by other psychological mechanisms [37].

Unique to the present study, risk factors for arthritis symptoms were examined separately in mid-age women and in older women, which allowed us to detect age-related differences in the association between physical activity and stiff or painful joints. Other strengths of this study were that it included a large population-based sample of women and used a prospective design. Women in each cohort who reported stiff or painful joints 'sometimes' or 'often' at T1 were excluded to reduce the possibility of reverse causation (that is, women became inactive because they had stiff or painful joints). Other strengths were that we used a validated and reliable measure of physical activity [25-27] and that we provided evidence of the predictive validity for our stiff and painful joints measure against self-reported physician-diagnosed arthritis and physical functioning.

A major limitation of this study was that all the data were self-reported. We did not have radiological or clinical measures, so we chose to focus on symptoms rather than on clinically diagnosed arthritis. This provided the opportunity to include women who may not have yet sought medical care or not yet been diagnosed with the problem. While it could be argued that the question about symptoms lacks specificity and sensitivity when compared with more objective measures, other researchers have shown that reporting these symptoms is associated with decreased ability to conduct functional tasks and with disability [38]. Previous studies have also shown that people underreported confirmed diagnoses when asked to report physician-diagnosed osteoarthritis, indicating that the burden of arthritis in the population has been underestimated [7,39].

Another limitation is the potential effect of participation bias on the results. Although the ALSWH included a fairly representative national sample of mid-age women and older women at the first data collection point [21], as with all prospective studies, there is continual attrition over time, with a tendency for more healthy women to remain in the cohort [40]. This 'healthy' participation bias was further exaggerated here by our inclusion of only women who did *not *report having stiff or painful joints 'sometimes' or 'often' at T1. While this was done to reduce the possibility of reverse causation (as described above), the original participation bias, together with the selection bias of women without joint pain or stiffness and exclusion of women with missing physical activity data, meant that our samples were more physically active than the general population of mid-age women and older women. The findings cannot, therefore, be generalized to all women in these age groups.

We were unable to examine factors associated with specific sites of the joint symptoms (for example, knee versus wrist), or about the year when the stiff or painful joint symptoms first developed, precluding the use of survival analysis or other procedures that require the exact duration of follow-up to be known. Finally, because few women in the ALSWH cohorts reported levels of physical activity that would be typically associated with 'athletic' training, we were unable to confirm findings from previous studies indicating that competitive sport and associated injuries might be involved in the development of osteoarthritis [8,10].

## Conclusion

The prevalence of arthritis in Australia is rapidly approaching that of cardiovascular disease [2]. As the cost to the Australian healthcare system of managing arthritis and its symptoms is likely to be greater than for other prominent health problems such as diabetes and asthma [2], the identification of physical inactivity as a potentially modifiable risk factor of incident stiff or painful joints among older women is important. Indeed, if preventive intervention strategies, such as increasing physical activity participation by even small amounts, could delay the onset and development of symptoms of arthritis, there could be considerable cost savings to the healthcare system and to older women themselves, not to mention reductions in pain and suffering caused by this often debilitating health problem.

## Abbreviations

ALSWH = Australian Longitudinal Study on Women's Health; BMI = body mass index; CI = confidence interval; OR = odds ratio; MET = metabolic equivalent value; SPJ = stiff or painful joints.

## Competing interests

The authors declare that they have no completing interests.

## Authors' contributions

KCH and YDM participated in the study conception and design, statistical analyses, interpretation of the data, and drafting of the manuscript. WJB participated in the study conception, study design, data acquisition, interpretation of the data, and drafting of the manuscript. All authors have read and approved the final manuscript.
